# Photodegradable Polyesters for Triggered Release

**DOI:** 10.3390/ijms131216387

**Published:** 2012-12-03

**Authors:** Cong Lv, Zhen Wang, Peng Wang, Xinjing Tang

**Affiliations:** State Key Laboratory of Natural and Biomimetic Drugs, the School of Pharmaceutical Sciences, Peking University, No. 38, Xueyuan Road, Beijing 100191, China; E-Mails: lvcong@bjmu.edu.cn (C.L.); wangzhen@bjmu.edu.cn (Z.W.); pengwang@bjmu.edu.cn (P.W.)

**Keywords:** photoactivation, polyester, nanoparticles, triggered release

## Abstract

Photodegradable polyesters were synthesized with a photolabile monomer 2-nitrophenylethylene glycol and dioyl chlorides with different lengths. These polymers can be assembled to form polymeric particles with encapsulation of target substances. Light activation can degrade these particles and release payloads in both aqueous solutions and RAW 264.7 cells.

## 1. Introduction

Smart polymers are a family of polymers that are responsive to external triggers [1–5], such as pH [6–9], specific enzymes [10,11], temperature [12–15], ultrasound [16,17] and light [18–23]. These polymers are capable of forming stimuli-sensitive carriers, including stimuli-sensitive micelles [24–26], liposomes [27–29], dendrimers [19,30–32], hydrogel [33–35] and microparticles [36–38] as smart drug delivery systems. Light as an external trigger is particularly attractive, since it can be remotely and accurately controlled, quickly switched and easily focused into specific areas with relatively high spatiotemporal resolution [39]. Photoresponsive diblock copolymers can be self-assembled into micelles with encapsulation of a target substance. Light triggered the cleavage of hydrophobic photosensitive blocks or crosslinkers to destabilize micelles for the release of target substances [40–41].

Particles formed with fully light-degradable polymers were developed for photo-triggered degradation of nanoparticles and burst release of payloads upon irradiation. Light-sensitive polymers containing photolabile and quinone-methide self-immolative moieties were synthesized and formulated into nanoparticles with the encapsulation of target substances by Almutairi [42–44]. In our previous work, photolabile polyurethanes were synthesized through bis(4-nitrophenyl)-1-(2-nitrophenyl) ethane-1,2-diyl dicarbonate and diamines [45]. In this paper, we simplified the syntheses of photolabile polymers with direct formation of polyesters by the coupling of 2-nitrophenylethylene glycol and dioyl dichlorides with different lengths. The polymers were able to assemble photodegradable nanoparticles via simple single emulsion and were demonstrated to respond to UV light irradiation fast with direct cleavage of the polymer backbone. The prepared nanoparticles were further characterized by scanning electron microscopy (SEM) and dynamic light scattering (DLS). To confirm the burst release, Nile red, a hydrophobic small mode molecule, was further encapsulated in particles via an emulsion technique (oil/water). A decrease in fluorescence intensity of Nile red-loaded particle solutions illustrated the release of the payload in 15 min. Cellular uptake and release of payload in cells were visualized with fluorescein diacetate-loaded particles.

## 2. Results and Discussion

### 2.1. Synthesis and Characterization of Photo-Degradable Polymers

**Scheme 1:**
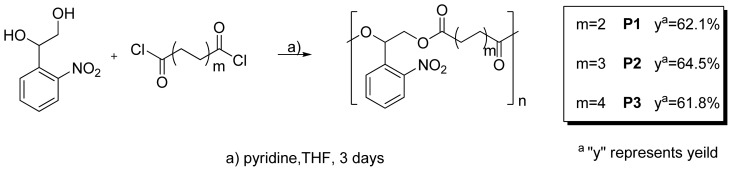
Synthesis of photodegradable polymers **P1**–**3**.

Three different polyesters **P1**–**3** were synthesized via step-growth polymerization of photolabile monomer 2-nitrophenylethylene glycol and dioyl chlorides with different lengths (Scheme 1). These polymers were then characterized by ^1^H-NMR to indicate the formation of polyesters with a 1-to-1 ratio of two units. Further characterization by GPC shows the degree of polymerization were around 11–13, according to the average molecular weight (*Mn*) of 3600, 4200, 3900 for **P1**–**3**, respectively (Table 1).

**Figure 1:**
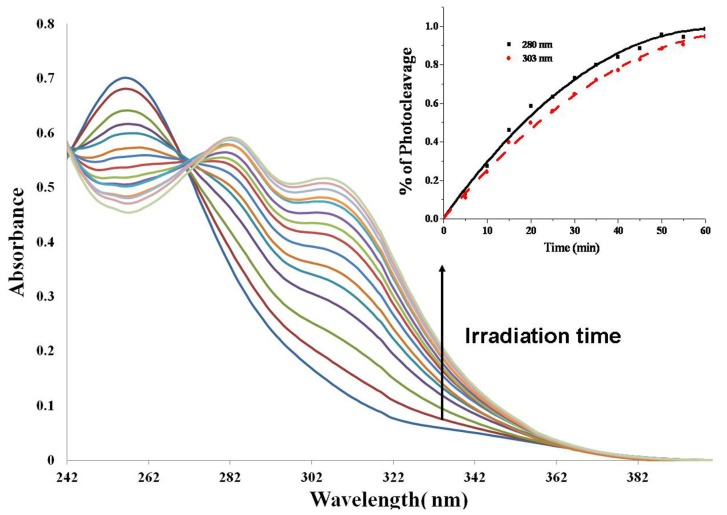
UV-Vis absorbance spectra of polymer **P3** upon photolysis at 365 nm (50 μg/mL).

To investigate the photocleavage of these polymers, UV-vis spectra of these polymers were monitored upon UV irradiation (Figures 1 and S1). Figure 1 shows the changes in absorbance spectra of **P3** in acetonitrile/H_2_O. With light irradiation (365 nm, 11 mW/cm^2^), the photolysis process of polymer **P3** was followed by noting the appearance of a shoulder absorption 303 nm and 280 nm, which are related to the absorption of byproduct 2-nitrosoacetophenone moiety [45,46]. The absorption spectra did not change any more after 60 min irradiation, which indicated that polymers can be fully photodegraded to small units around 60 min. We also checked the photocleavage of polymers by examining the proton signal Ph-*CH*-CH_2_ by ^1^H-NMR in **P3***d6*-DMSO solution with 10 min irradiation. We can find the 32% decrease of this signal after UV irradiation. Polymer samples irradiated with 15 min in THF were subject to GPC analysis. The average molecular weights of all the polymers were decreased, which also indicated the ability of photodegradation of these polymers as shown in Table 1 and Figure S2. The photo-degradation property of these polyesters into small fragments may be used to trigger the burst release of encapsulated substances from nanoparticles formed with these photodegradable polymers.

### 2.2. Nanoparticle Preparation and Characterization

Polymeric nanoparticles (**Np1**–**3**) were prepared using an oil/water emulsion technique with polyesters **P1**–**3**. The emulsion was formed through sonication of these polyester solutions in methylene chloride (DCM) with an aqueous phase containing polyvinyl alcohol as an emulsion stabilizer. These nanoparticles were further characterized by SEM and DLS. The particles were found to have average diameters of 221–234 nm with low polydispersity index according to DLS data (Table 2 and Figure S3). The surface charge of the particles was also determined by zeta-potential measurements (Table S1). All particles have a slightly negative zeta-potential due to the carboxylic acid group at two terminals of polymers.

**Figure 2:**
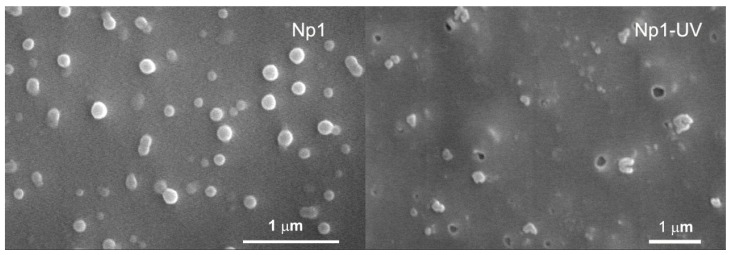
SEM images of **Np1**, left without UV irradiation, right with UV irradiation.

SEM results showed that these polymers formed nanoparticles with spherical morphology and similar particle sizes in comparison to DLS results. The photo-degradation behaviors of nanoparticles were also confirmed by SEM. Figure 2 shows that typical SEM images of **Np1**. Without light irradiation, particles were in spherical shapes. However, lots of spherical particles crashed upon light activation. Similar observations also happened for the other nanoparticles (Figure S4).

**Figure 3:**
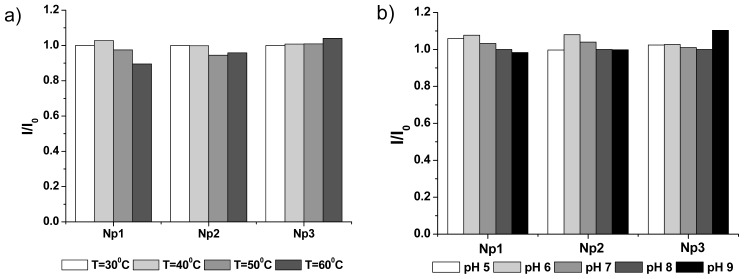
Relative stability of nanoparticles (**a**) at different temperatures and (**b**) in the buffers with different pH values. The Nile red fluorescence intensity of nanoparticle solutions at 30 °C, and pH 7.0 was used as the standard.

High stability of polymeric nanoparticles before triggering is greatly favored for an ideal drug carrier, for preventing drugs from leaking during circulation in blood. To study the stability of nanoparticles themselves in solutions, Nile red was encapsulated into these polymeric nanoparticles. This small molecule was chosen because of its excellent photostability and dramatic change of fluorescence intensity in hydrophobic particle core and outside aqueous solution. Fluorescence intensity of Nile red-loaded nanoparticle solutions was recorded in buffers with different pH, or incubated at different temperatures, as shown in Figure 3. As we can see, nanoparticles are still quite stable without the loss of Nile red fluorescence for all nanoparticles with the temperature up to 60 °C. By changing the pH value of buffers from 4 to 9, the variation in Nile red fluorescence intensity is relatively small. These results further confirmed that all these photolabile nanoparticles were relatively stable before light irradiation. Furthermore, the stability of nanoparticles was also evaluated with a long time standing. After storing at room temperature for seven days, Nile red fluorescence intensity of particle solutions in different buffers almost did not change. Np-3 particle solution (pH = 7) showed a little bit of decrease of Nile red fluorescence intensity. This may be due to a long hydrophobic alkyl group, which caused the more hydrophobic surface of nanoparticles and easier aggregation and precipitation of particles. With gentle mixing, Nile red fluorescence intensity of Np-3 solution was almost recovered, which showed that no obvious leak of encapsulated Nile red happened. In addition, phototriggered Nile red releasing behaviors of particle solutions are similar upon seven days standing at room temperature, based on Nile red fluorescence measurement (Figure S5).

### 2.3. Photoresponsive Behaviors of Nanoparticles

**Figure 4:**
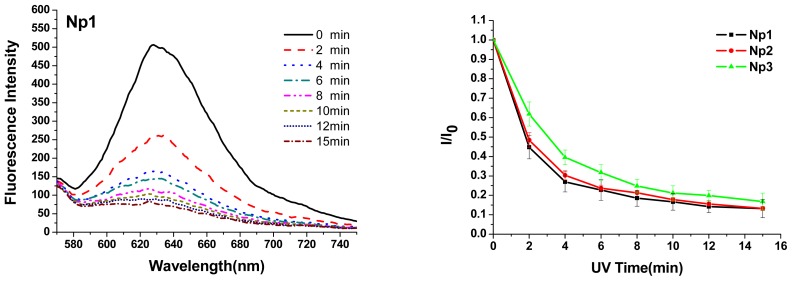
(**a**) Fluorescence spectra of Nile red-loaded **Np1** solution upon light irradiation (365 nm, 11 mW/cm^2^) with excitation wavelength at 550 nm; (**b**) Plots of normalized fluorescence intensity of Nile red at 630 nm *vs*. irradiation time for aqueous solutions of **Np1**–**3**.

To evaluate the photoresponsive behaviors of nanoparticles, fluorescence spectra of these Nile red-loaded particle solutions were recorded, and the decrease in Nile red fluorescence intensity at 630 nm with the excitation wavelength of 550 nm was monitored. Upon 15 min irradiation, fluorescence intensity of all Nile red-loaded particles dropped by 83%–87% for **Np1**–**3**, indicating the dramatic breakage of nanoparticles and the release of payload, even though the polymers may not be fully photodegraded to small units (Figures 4 and S6). On the other hand, 2 min irradiation with 365 nm light triggered more than a 50% drop of fluorescence intensity, indicating the burst release of Nile red from all nanoparticles. These results demonstrated that full degradation of photolabile polymers are not necessary in order to trigger the release of payload from the particles formed with these polymers.

**Figure 5:**
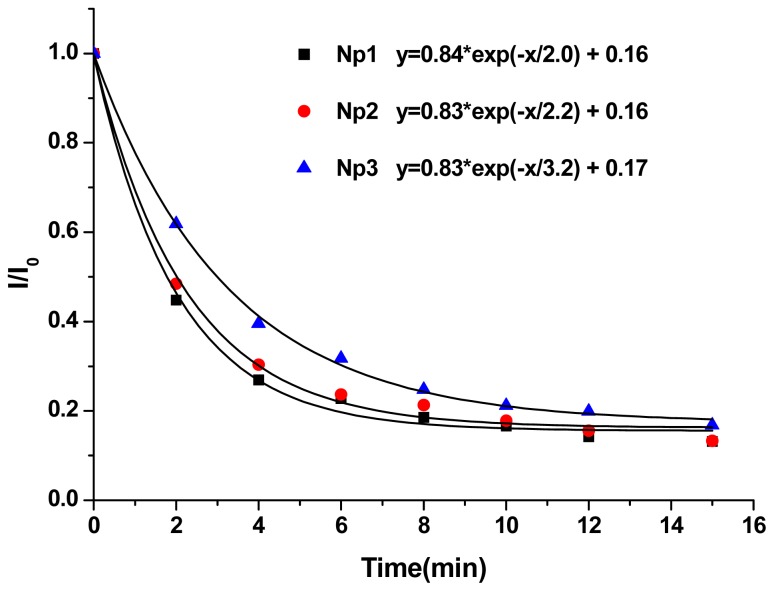
Plots of normalized fluorescence *vs*. irradiation time and curve fitting for Nile red loaded particle Np1–3 aqueous solutions exposed to UV light (365 nm, 11 mW/cm^2^).

Kinetics of photo-triggering Nile red release is also studied. By fitting the data of relative Nile red fluorescence intensity *I*/*I*_0_*vs*. irradiation time (min), the characteristic time τ was obtained through the following equation (Figure 5). Here, (I/I_0_)_m_ is the achievable minimum of the normalized fluorescence, and τ is the characteristic time at which the fluorescence intensity decreases to 36.8% (1/*e*). The results displayed τ for **Np1**–**3** was 2.0, 2.2 and 3.3 min, respectively.

$$I/I_{0}={\left(I/I_{0}\right)}_{\text{m}}+[1-{\left(I/I_{0}\right)}_{\text{m}}]\;\text{exp}(-t/\tau )$$

By comparing **Np1**–**3**, the encapsulation ability was similar based on the fluorescence intensity of Nile red, while the longer hydrophobic dioyl chloride chains show a slower rate of triggered release. This is probably due to stronger hydrophobic chain-chain interaction of longer dioyl subunits, which can hold Nile red tightly and release it slowly.

### 2.4. Nanoparticle Uptake and Release in Cell Study

**Figure 6:**
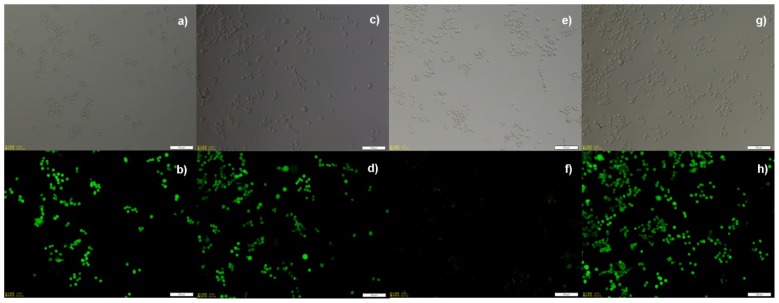
Fluorescence microscopy images of RAW 264.7 cells incubated with (**a**, **b**) free FDA; (**c**, **d**) free FDA followed by 15 min light irradiation; (**e**, **f**) FDA-loaded **Np1** for 4 h and (**g**, **h**) FDA-loaded **Np1** for 4 h followed by 15 min light irradiation. The concentration of FDA-loaded **Np1** was 25 μg/mL. (scale bar = 100 μm)

In the study of nanoparticle uptake and triggered release in macrophages, a mouse macrophage cell line RAW 264.7 was used. Fluorescein diacetate (FDA) was entrapped into nanoparticles as a model fluorophore due to the fact that FDA itself is nonfluorescent, while upon uptake by cells, intracellular esterases hydrolyze diacetate groups, producing a highly fluorescent product (fluorescein). With FDA encapsulation, cellular uptake and triggered release of nanoparticles were visualized directly by fluorescence microscopy. RAW264.7 cells were first incubated with FDA-loaded nanoparticles for 4 h, and then were thoroughly washed with PBS buffer. Figure 6 shows fluorescent images of macrophages under different conditions. Figure 6a,b are the images of control experiments with the addition of only FDA, which lights up cells in less than 10 min due to the quick hydrolysis of FDA by esterases in cells. Figure 6e, f are images of cells uptaken with FDA-loaded nanoparticles **Np1** without light activation. As we expected, nanoparticles are stable in cells, and there is almost no fluorescence emission. Figure 6g, h clearly show the cells light up with hydrolyzed FDA upon light activation. In comparison to the control, FDA-loaded photolabile nanoparticles must have been photodegraded, and the breakage of polymers triggers the release of FDA, followed by the hydrolysis with intracellular esterases. In addition, **Np2** and **Np3** were also incubated with RAW 264.7 cells, and similar observations were demonstrated in Figure S7. Cytotoxicity of these nanoparticles was also investigated by incubating cells with various concentrations of nanoparticles for 24 h. (Figure S8). The MTT assay results show that blank particles and their degradation products have similar toxicity over the range of concentrations up to 1000 μg/mL, which indicated that long-term toxicity of particles formed by these polyesters may be due to the low molecular weight and/or the cellular hydrolysis of these polyesters.

## 3. Experimental Section

All commercially obtained solvents and reagents were used without further purification, except as noted below. Tetrahydrofuran (THF), dichloromethane (DCM) and pyridine were distilled over calcium hydride prior to use. Sebacoyl dichloride and adipoyl dichloride were purchased from TCI, Shanghai. Suberoyl dichloride was purchased from Alfa Aesar. Nile red was purchased from J&K Chemicals. Polyvinyl alcohol (PVA with average molecular weight of 16,000 Da) was purchased from Acros Organics.

^1^H NMR and ^13^C NMR spectra were recorded on a Bruker 400 MHz NMR spectrometer. Reported chemical shifts (ppm) are relative to CDCl_3_ and coupling constants are reported in Hz. UV-vis spectra were obtained using DU800 UV-vis spectrophotometer. Fluorescence spectra were performed on Cary Eclipse fluorometer. For Nile red, an excitation wavelength of 550 nm was used, and emission spectra were recorded from 570 and 750 nm. The size in diameter and zeta-potential of nanoparticles were determined using dynamic light scattering (DLS) at 25 °C using a Zetasizer Nana-ZS from Malvern Instruments (Cumulant method). Scanning electron microscopy (SEM, Hitachi S-4300) was used to characterize nanoparticles. Cellular uptake of the particles was visualized with an inverted fluorescence microscope (OLYMPUS, IX81). The toxicity of particles was detected with FlexStation 3 (Molecular Devices). B-100SP (UVP, LLC) was used as light resource for irradiation (365 nm, 11 mW/cm^2^).

### 3.1. Chemical Synthesis

Monomer 1 (0.92 g, 5 mmol) and dioyl chloride (5 mmol) were dissolved in 15 mL THF under nitrogen, and pyridine (2 mL, 25 mmol) was added to the reaction mixture dropwise on ice. The polymerization was allowed to proceed three days at room temperature. The reaction mixture was extracted with DCM, washed with aqueous brine and dried over anhydrous Na_2_SO_4_. The organic layer was concentrated to 2 mL and precipitated into 20 mL of cold EtOH, yielding polymer. The short oligomers were removed by repeated precipitation of these polymers into cold EtOH. The characterization of polymers was as following (Figure S9).

**P1**, Yield: 62.1%.

^1^H-NMR (400 MHz, CDCl_3_) δ 7.99 (br d, 1H), 7.63 (br d, 2H), 7.49 (br s, 1H), 6.50 (br s, 1H), 4.46 (br d, CH*CH_2_*O, 2H), 2.30 (br, CO*CH_2_*, 2H), 2.37 (br, CO*CH_2_*, 2H), 1.59 (br, CH_2_*(CH_2_*)_2_CH_2_, 4H).

^13^C-NMR (100 MHz, CDCl_3_) δ 172.68 (C=O), 171.95 (C=O), 148.18 (Ar), 133.74 (Ar), 132.63 (Ar), 129.45 (Ar), 128.54 (Ar), 124.96 (Ar), 69.40 (CH), 65.10 (CH_2_), 33.68 (CH_2_), 24.19 (CH_2_).

**P2**, Yield: 64.5%.

^1^H-NMR (400 MHz, CDCl_3_) δ 8.00 (br d, 1H), 7.64 (br s, 2H), 7.49 (br s, 1H), 6.52 (br s, 1H), 4.46 (br d, CH*CH_2_*O, 2H), 2.29 (br, CO*CH_2_*, 2H), 2.35 (br, CO*CH_2_*, 2H), 1.57 (br, CH_2_*CH_2_*(CH_2_)_2_*CH_2_*CH_2_, 4H), 1.28 (br, CH_2_CH_2_(*CH_2_*)*_2_*CH_2_ CH_2_, 4H).

^13^C-NMR (100 MHz, CDCl_3_) δ 173.11 (C=O), 172.34 (C=O), 148.24 (Ar), 133.63 (Ar), 132.82 (Ar), 129.42 (Ar), 128.54 (Ar), 124.98 (Ar), 69.32 (CH), 65.08 (CH_2_), 34.13 −33.99 (CH_2_), 28.76 (CH_2_), 24.66 (CH_2_).

**P3**, Yield: 61.8%.

^1^H-NMR (400 MHz, CDCl_3_) δ 7.99 (br d, 1H), 7.63 (br s, 2H), 7.48 (br s, 1H), 6.52 (br s, 1H), 4.48 (br d, CH*CH_2_*O, 2H), 2.29 (br, CO*CH_2_*, 2H), 2.35 (br, CO*CH_2_*, 2H),1.57 (br, CH_2_*CH_2_*(CH_2_)_4_*CH_2_*CH_2_, 4H), 1.25 (br, CH_2_CH_2_(*CH_2_*)*_4_*CH_2_ CH_2_, 8H).

^13^C-NMR (100 MHz, CDCl_3_) δ 172.92 (C=O), 172.11 (C=O), 147.89 (Ar), 133.24 (Ar), 132.54 (Ar), 129.03 (Ar), 128.21 (Ar), 124.63 (Ar), 68.96 (CH), 64.71 (CH_2_), 33.90 (CH_2_), 33.76 (CH_2_), 28.80 (CH_2_), 24.51 (CH_2_).

### 3.2. Polymer Characterization and Photodegradation Study

The molecular weight of these polymers was determined using gel permeation chromatography (GPC). GPC measurement was performed with a Waters 1515 HPLC pump, a Waters 2414 refractive index detector and a combination of Styragel HT-2, HT-3 and HT-4, whose effective molar mass ranges are 100–10,000, 500–30,000 and 5000–600,000, respectively. Tetrahydrofuran was used as the eluent at a flow rate of 1.0 mL/min at 35 °C. Polystyrenes with narrowly distributed molecular weights were used as standards for calibration. Polymers (2–5mg) were dissolved in THF (1 mL, HPLC grade), and the solutions standing overnight were injected to GPC without filtrating.

The degradation of these polymers was studied by UV-vis absorption spectra. 0.05 mg/mL solution of polymers in acetonitrile/H_2_O (9/1) was irradiated for the specified periods of time, and UV-vis absorption spectra were recorded after each irradiation. The degraded fragments of polymers were analyzed by GPC in THF solutions before or after 15 min light irradiation.

### 3.3. Preparation of Nanoparticles from Polyesters **P1**–**3**

Nanoparticles **Np1**–**3** were prepared according to the literature [45]. **P1**–**3** (20 mg) dissolved in 2 mL DCM were added to 40 mL PVA solution (1%), followed by stirring at 1000 rpm for 10 min. Emulsification of the above mixture was achieved by 5 min sonication on ice at 50 W. The suspension was further vigorously stirred using a magnetic stirrer, allowing organic solvent to evaporate overnight. Nanoparticles were recovered by ultracentrifugation at 18,000 rpm (~30,000× *g*) for 20 min and washed twice with water.

Nile red-loaded nanoparticles were prepared as described above for blank particles. A solution of polyesters in DCM containing 0.25 mg/mL Nile red was emulsified with an aqueous phase containing PVA by sonication. After evaporation of DCM, nanoparticles were collected by ultracentrifugation according to the above same protocol.

### 3.4. Nanoparticle Characterization and Degradation Study

Nanoparticles were characterized by scanning electron microscopy (SEM) using Nanoscope IIIa. The particles were dispersed in water, and then 5 μL particle solution was dripped onto a silicon wafer. After drying, particles were sputter coated with Aurum. Particle size distributions and average particle diameters were determined by dynamic light scattering using a Zatasizer Nano ZS (Malvern Instruments Ltd., Malvern, UK). Particles were suspended in water and the results are presented as an intensity distribution.

### 3.5. Light-Triggered Release Study

To study the light-controlled release of guest molecules in particles, Nile red was encapsulated into nanoparticles. Solutions of **Nps** in water were first irradiated for the specific periods of time, and fluorescence emission spectra of Nile red with the excitation wavelength at 550 nm were recorded after each irradiation. The concentrations of particle solutions were 2 mg/mL.

### 3.6. Particles Cytotoxicity Study

*In vitro* cytotoxicity of blank particles was evaluated by MTT assay with RAW 264.7 cells. Cells suspension in culture medium (DMEM + 10% FBS) with the concentration of 1 × 10^5^ cells/mL were seeded in 96-well plates, and incubated at 37 °C humidified atmosphere with 5% CO_2_ for 24 h. Cells were treated with various amounts of particles (1.6–1000 μg/mL) and incubated for 24 h. Each well was given 10 μL of MTT solution (5 mg/mL) and was incubated for 4 h. Three linked lysis solution (150 μL) was then added to cells to dissolve the resulting formazan crystals. After another 12 h of incubation, the absorbance at 570 nm was measured using Molecular Devices (Flexstation 3). The cell viability was determined by comparing the absorbance of particle-treated cells to that of control cells.

The cytotoxicity of the degraded blank particles and the influence of UV light were also studied. The aqueous solutions of particles were irradiated for 15 min, and the cell toxicity of the resulting degraded materials was tested as described above for the particles.

### 3.7. Nanoparticle Uptake and Release in Cell Study

To investigate nanoparticle uptake by cells and triggered release in cells, fluorescein diacetate (FDA) was encapsulated in nanoparticles. FDA-loaded nanoparticles were further incubated with RAW 264.7 macrophage cells for 4 h. After incubation, cells were washed five times with PBS buffer and were irradiated with 365 nm UV light for 15 min. The prepared samples before and after UV irradiation were then imaged on a fluorescence microscopy.

## 4. Conclusions

A family of photodegradable polyesters **P1**–**3** were synthesized and characterized by ^1^H-NMR, ^13^C-NMR and GPC, and their photolysis properties were studied by UV spectroscopy. The photodegradable polymers can be assembled into nanoparticles with encapsulation of water-insoluble drugs for delivery. The formation and photodegradation of nanoparticles (**Np1**–**3**) were characterized by DLS, SEM and Nile red fluorescence experiments, respectively. The results showed that particles formed with these polymers are quite stable in aqueous solutions with different pH buffers and at elevated temperatures. Light can trigger the cleavage of particles and the release of encapsulated substances with the efficiencies up to 83%–87% based on Nile red fluorescence intensity upon 15 min light irradiation. Full photo-degradation of polymers is not necessary for triggered release of encapsulated substances from these polymeric particles. Nanoparticle uptake by RAW 264.7 and light-triggered burst release in cells were observed by fluorescence emission of hydrolyzed fluorescein diacetate (FDA) upon photo-induced degradation of FDA loaded nanoparticles.

## Supplementary Information



## Figures and Tables

**Table 1:** GPC data of **P1**–**3** before and after 15 min UV irradiation, *MW* is calculated according to polystyrene standard.

Polymer	No. UV			Degree of polymerization	UV		
	
	*Mn* (Da)	*Mw* (Da)	PDI		*Mn* (Da)	*Mw* (Da)	PDI
P1	3600	7500	2.08	12.2	1000	1700	1.70
P2	4200	6200	1.49	13.0	1100	1700	1.60
P3	3900	5500	1.40	11.2	1200	2200	1.74

**Table 2:** Size distribution and zeta-potential of blank particles **Np1**–**3**.

Nps	Size (nm)	PDI	Z-P
Np-1	221.2	0.14	−27.9
Np-2	232.7	0.16	−26.7
Np-3	234.3	0.13	−25
