# Supplementary material for: Photodegradable Polyesters for Triggered Release
Source: Int J Mol Sci. 2012 Dec 3;13(12):16387–99. doi: 10.3390/ijms131216387 (PMC3546696; doi:10.3390/ijms131216387)

Supporting Information

Figure S1. UV-Vis absorbance spectra of polymer **P1–2** upon photolysis at 365 nm (50 μg/mL).

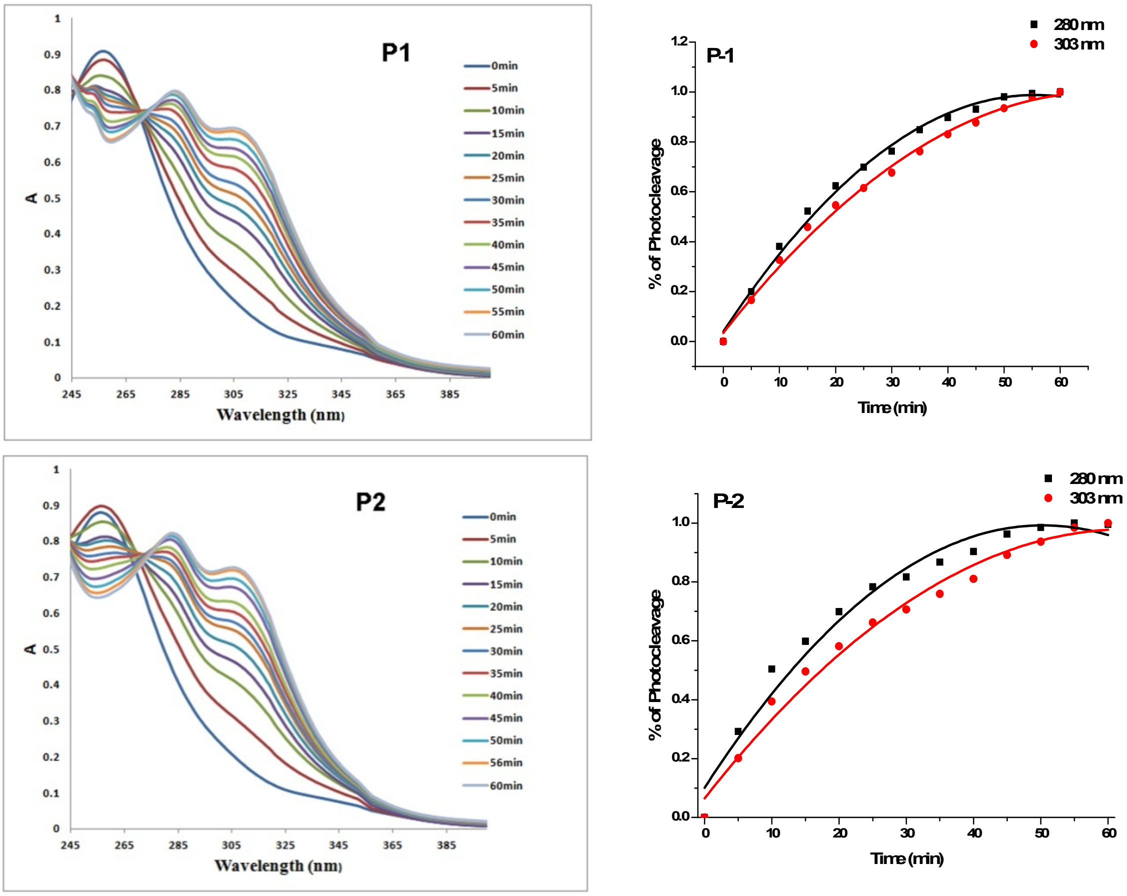

Figure S2. GPC traces of **P1**, **P2** and **P3** before and after 15 min light irradiation.

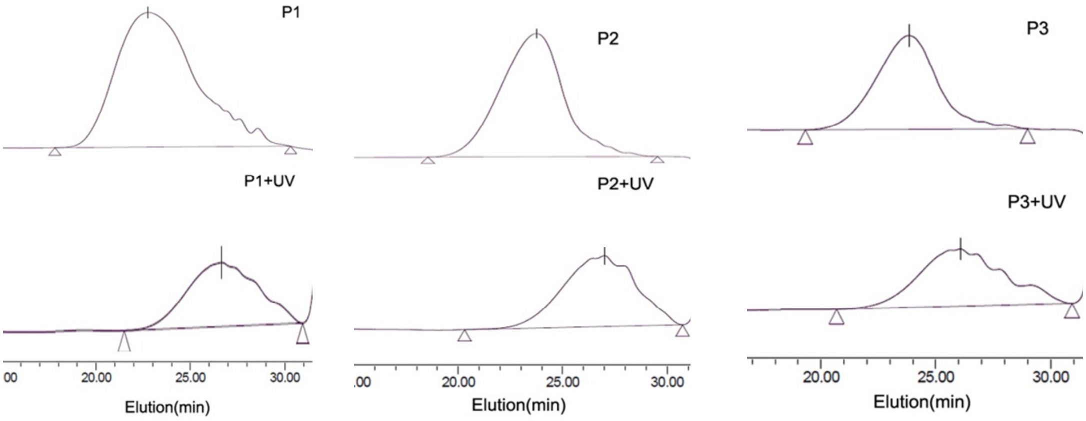

Figure S3. DLS distribution of Np1, Np2, Np3.

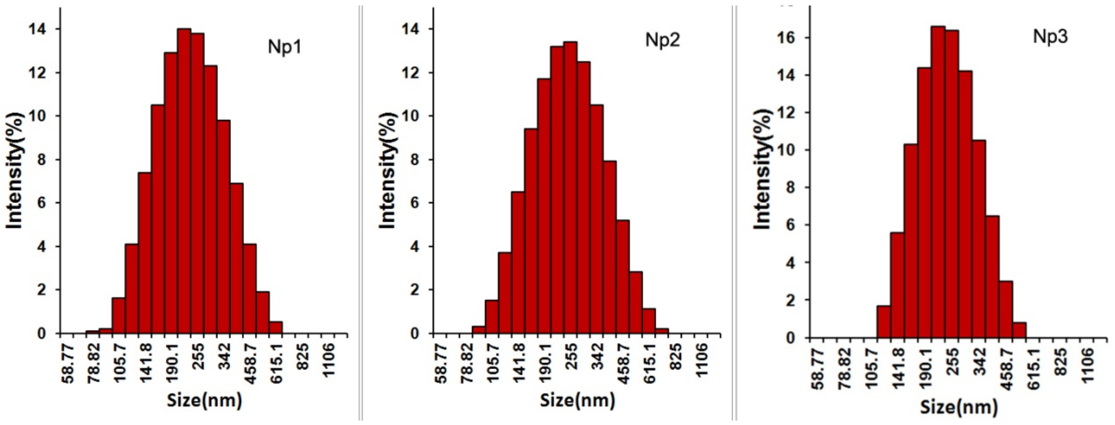

Figure S4. SEM images of **Np2** and **Np3**, left without UV irradiation, right with UV irradiation.

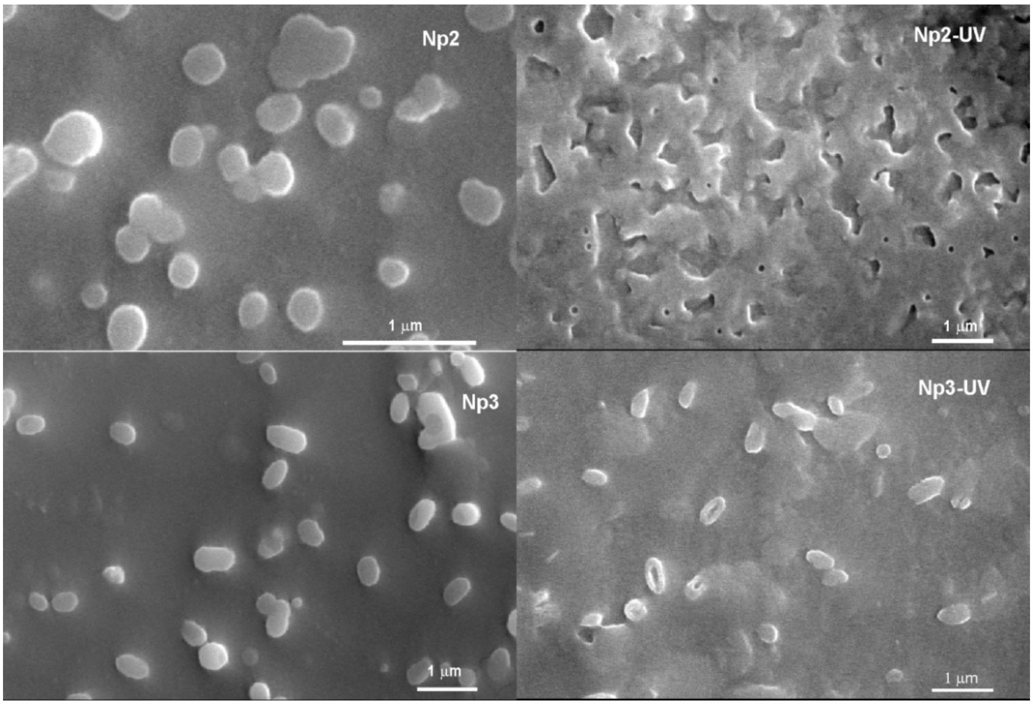

Figure S5. (**A**) Stability of Nile red-loaded nanoparticle aqueous solutions (pH = 7) and phototriggered Nile red releasing of particles upon 7 days standing at room temperature based on Nile red fluorescence measurement. (**B**) Stability of Nile red-loaded nanoparticle aqueous solutions in different buffers (pH = 5 and 9) in 7 days.

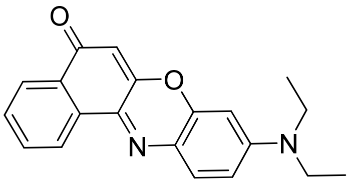

Nile red

Figure S5. *Cont.*

(A)

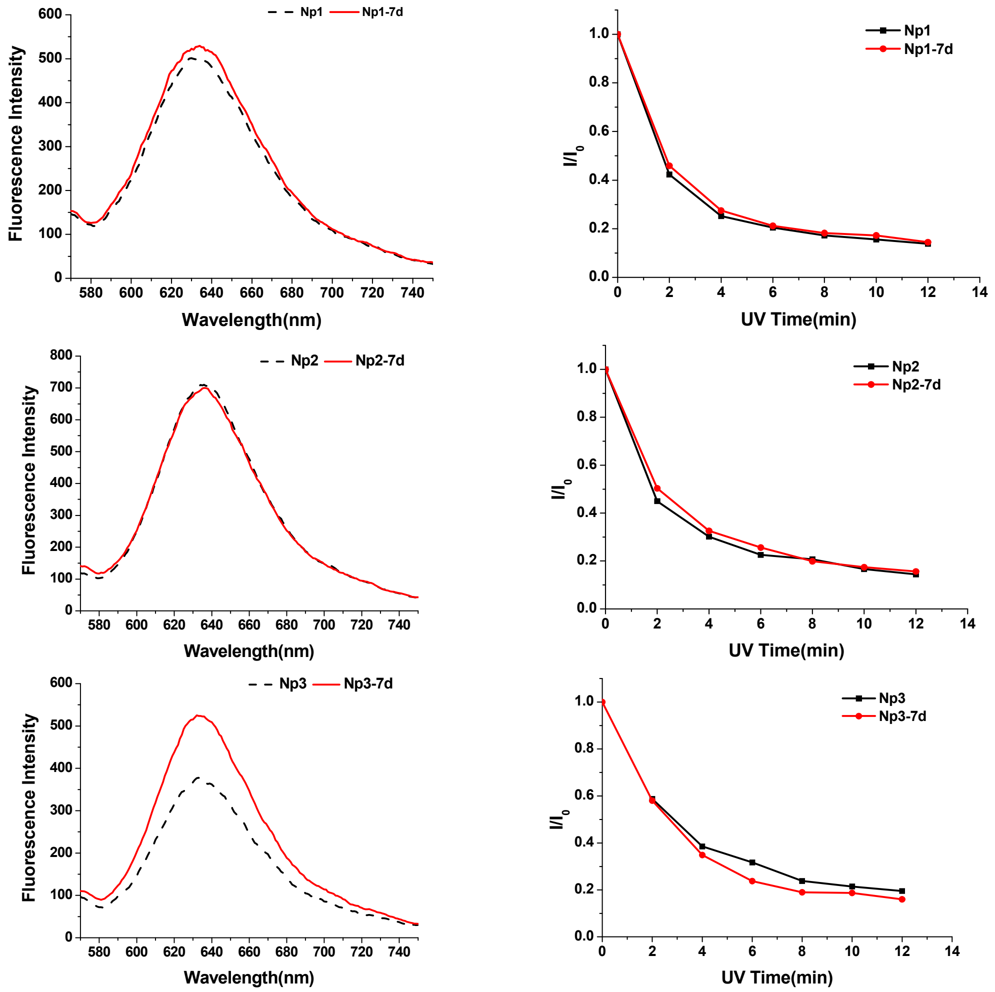

(B)

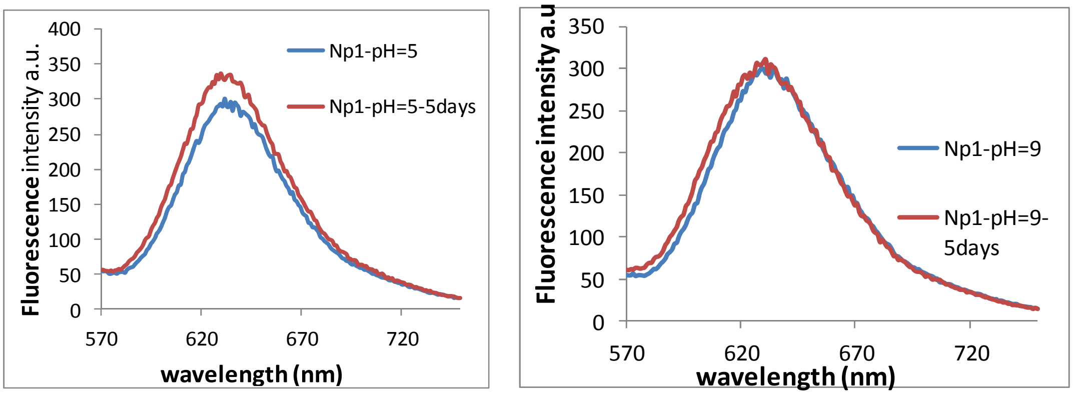

Figure S5. *Cont.*

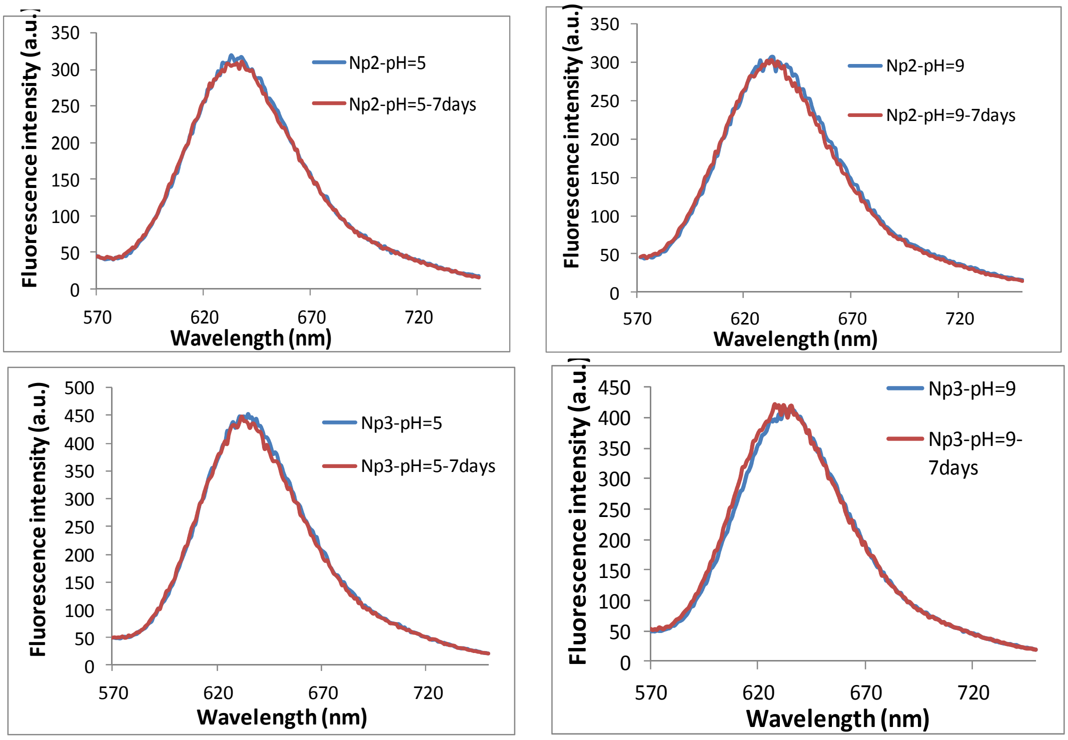

Figure S6. Fluorescence spectra of Nile red loaded nanoparticle **Np2** and **Np3** aqueous solutions upon light irradiation (365 nm, 11 mW/cm^2) with the excitation wavelength at 550 nm.

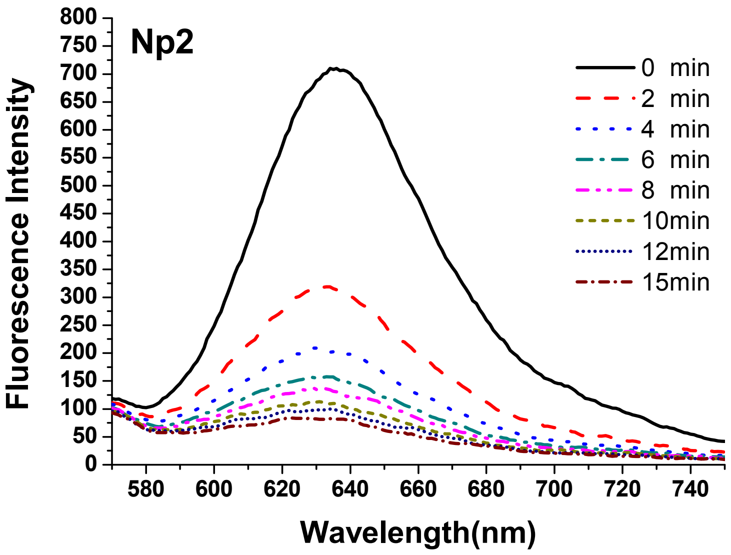

Figure S6. *Cont.*

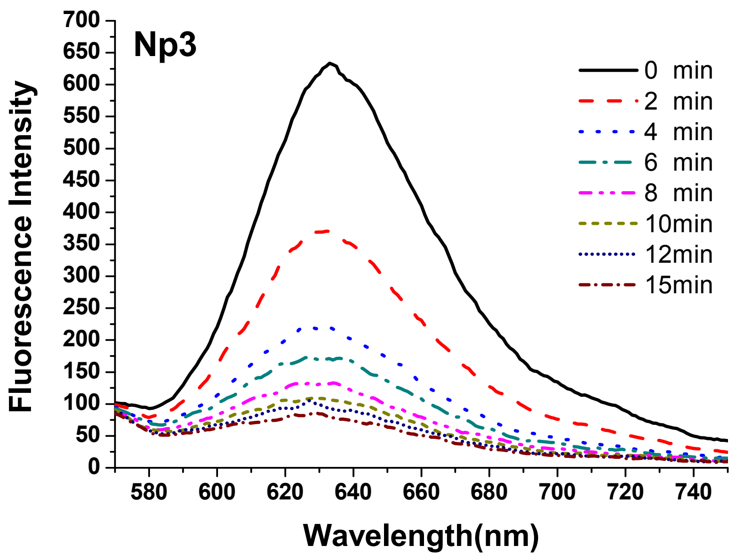

Figure S7. Fluorescence microscopy images of RAW 264.7 cells incubated with (**a**, **b**) FDA-loaded **Np2** for 4 h, (**c**, **d**) FDA-loaded **Np2** for 4 h followed by 15 min light irradiation, (**e**, **f**) FDA-loaded **Np3** for 4 h, and (**g**, **h**) FDA-loaded **Np3** for 4 h followed by 15 min light irradiation. (scale bar = 100 μm)

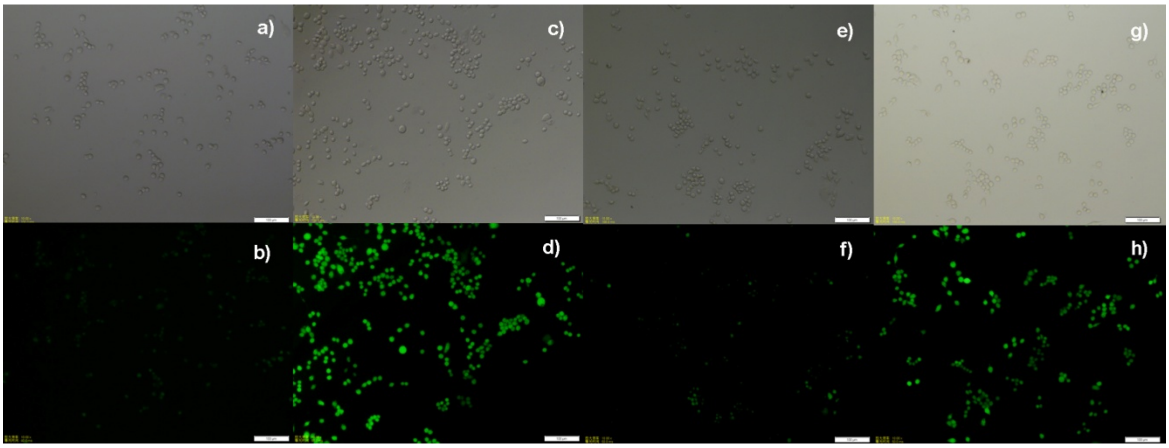

Table S1. Size distribution and zeta-potentials of FDA-Loaded **Np1–3**.

Nps	Size (nm)	PDI	Z–P
Np-1	191.6	0.10	−17.5
Np-2	204.1	0.25	−19.6
Np-3	198.0	0.13	−14.3

Figure S8. Cell toxicity of **Np1–3** measured by incubation with RAW 264.7 cells for 24 h. The viability of cells incubated with nanoparticles and irradiated nanoparticle residues was normalized to cells cultured without nanoparticles.

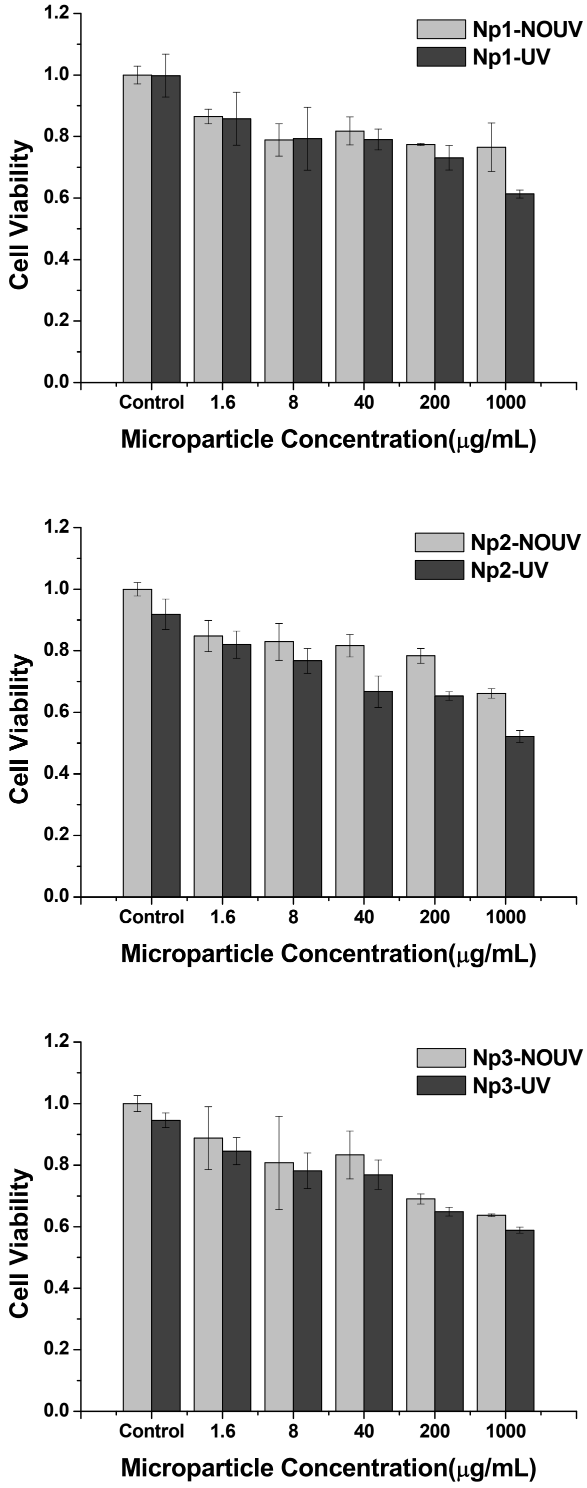

Figure S9. NMR spectra of polymer **P1**, **P2** and **P3**.

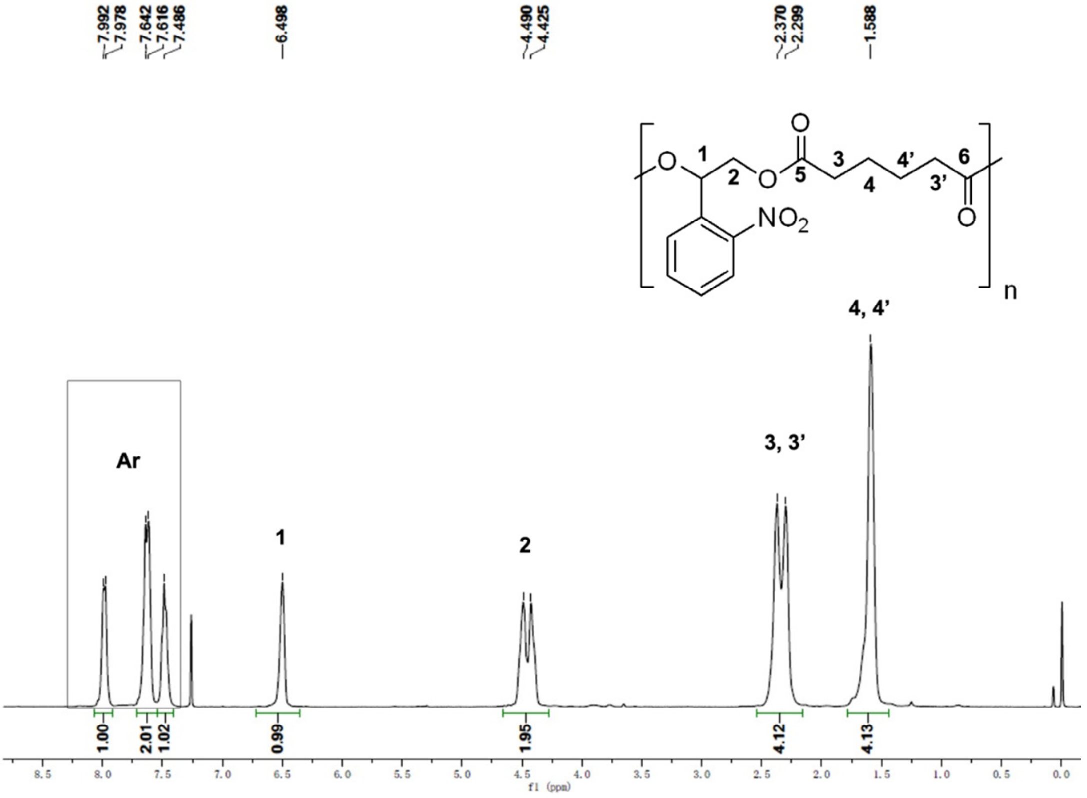

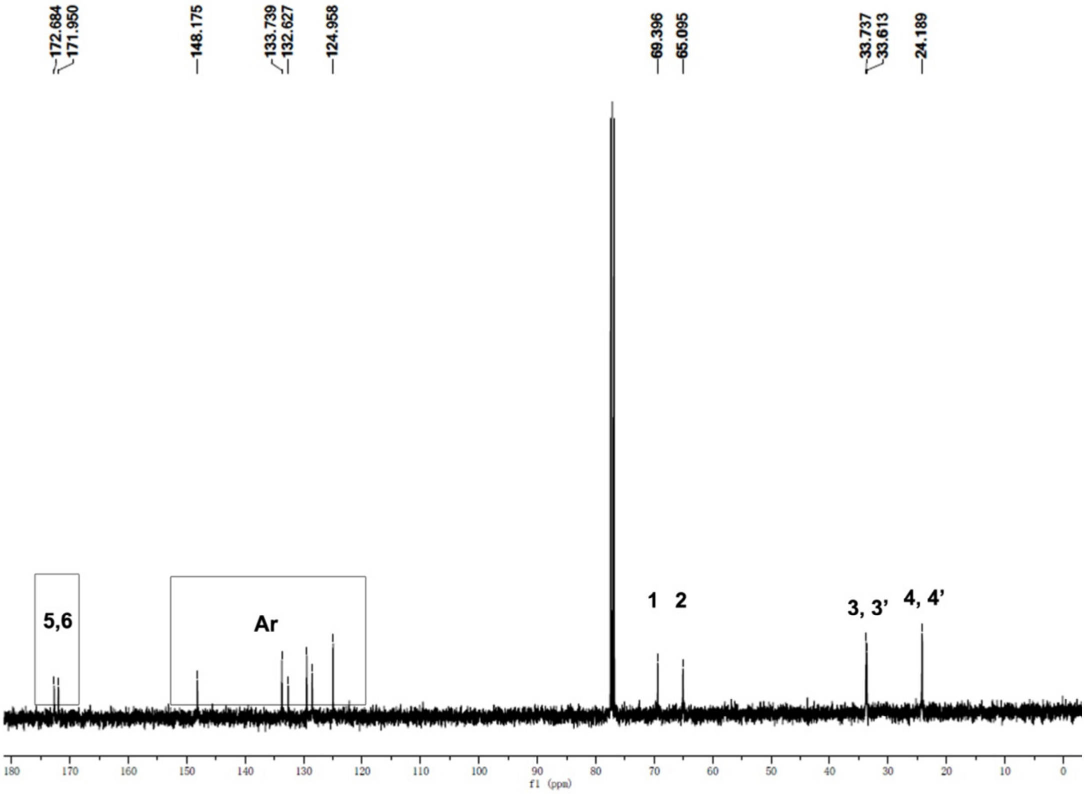

Figure S9. *Cont.*

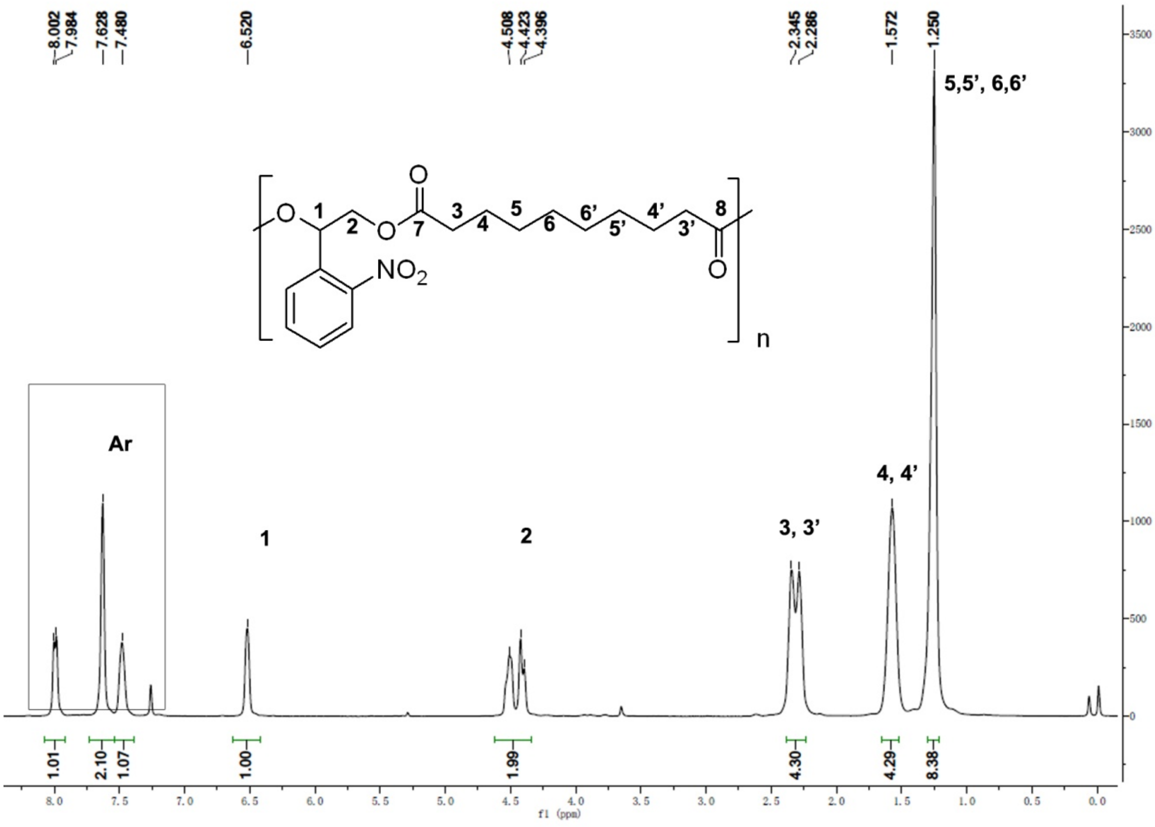

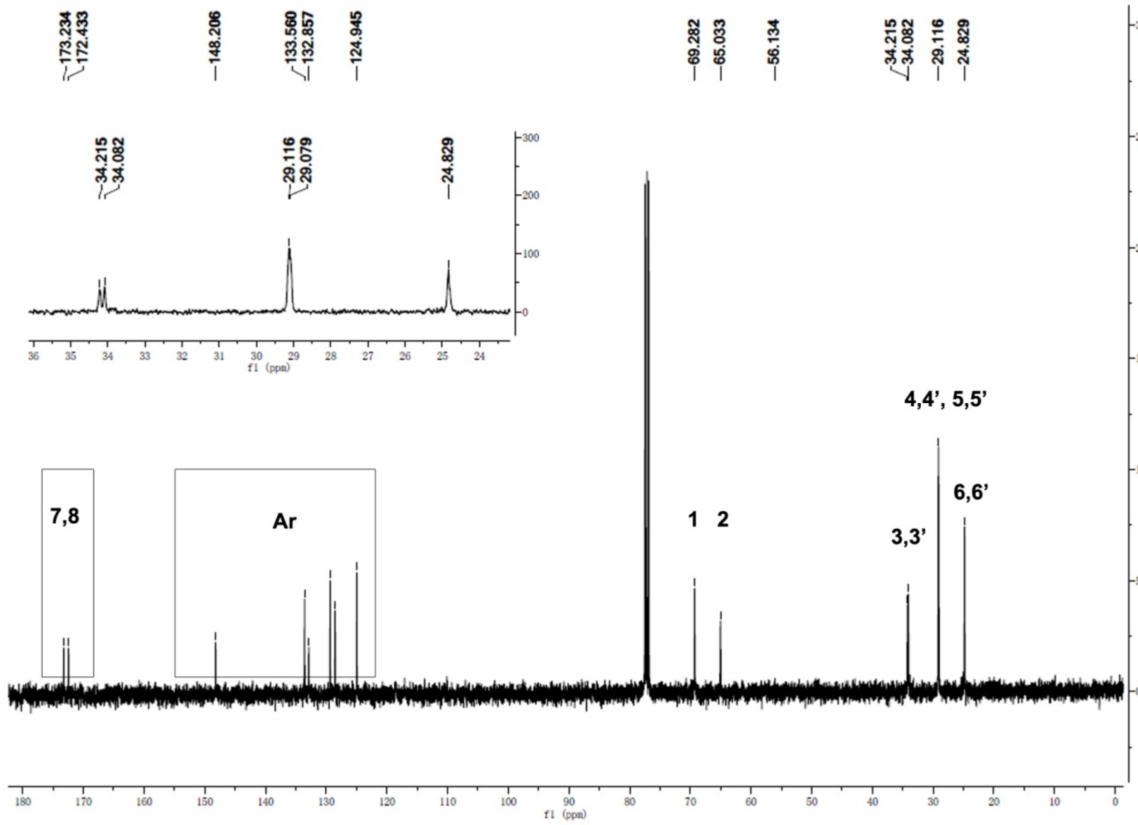

Figure S9. *Cont.*

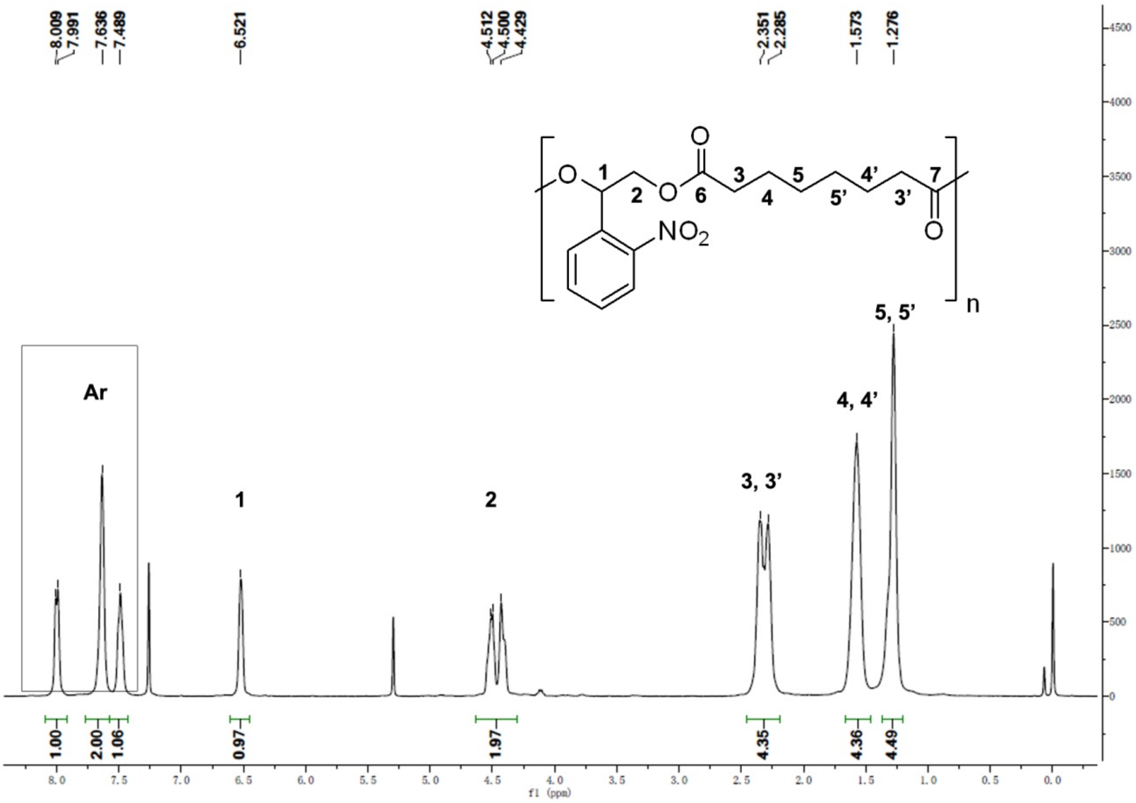

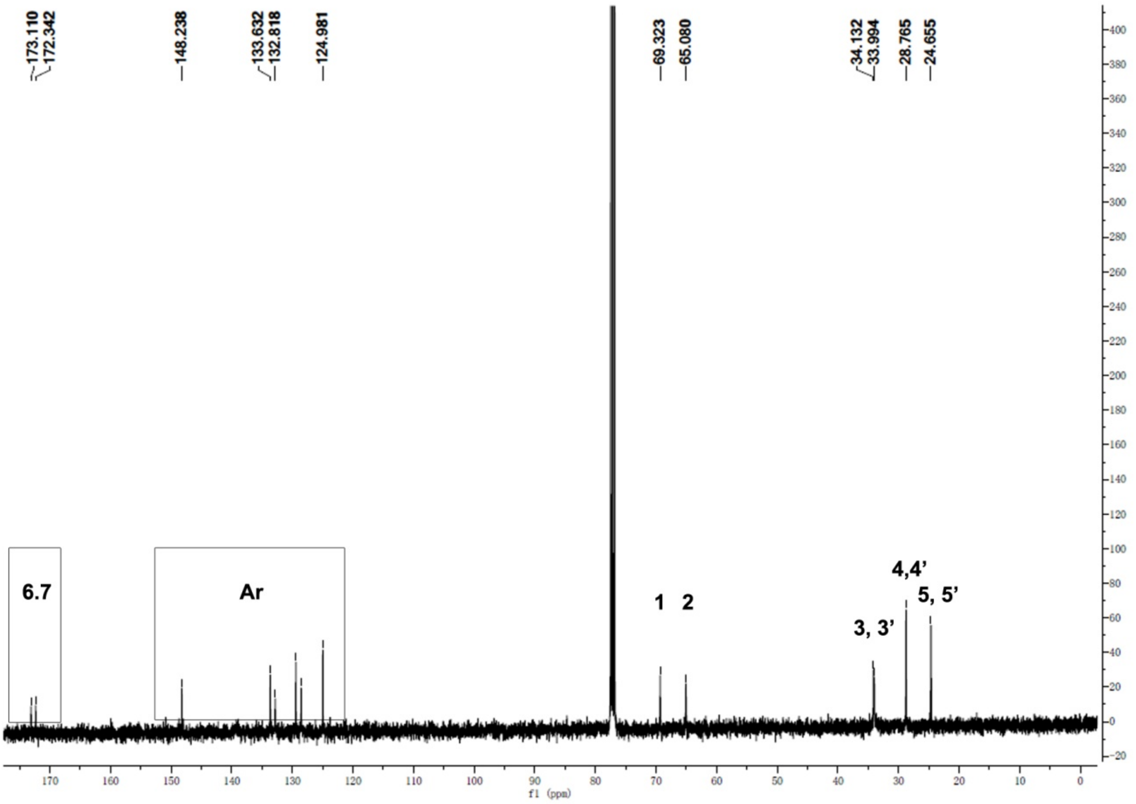

Supplement: Supplementary file 1 [file ijms-13-16387-s001.pdf]
